# Application of a pyramid pooling Unet model with integrated attention mechanism and Inception module in pancreatic tumor segmentation

**DOI:** 10.1002/acm2.14204

**Published:** 2023-11-08

**Authors:** Zhiwei Zhang, Hui Tian, Zhenshun Xu, Yun Bian, Jie Wu

**Affiliations:** ^1^ School of Health Science and Engineering University of Shanghai for Science and Technology Shanghai China; ^2^ Department of Radiology Changhai Hospital The Navy Military Medical University Shanghai China

**Keywords:** attention mechanism, deep learning, image segmentation, pancreatic cystic neoplasms

## Abstract

**Background:**

The segmentation and recognition of pancreatic tumors are crucial tasks in the diagnosis and treatment of pancreatic diseases. However, due to the relatively small proportion of the pancreas in the abdomen and significant shape and size variations, pancreatic tumor segmentation poses considerable challenges.

**Purpose:**

To construct a network model that combines a pyramid pooling module with Inception architecture and SE attention mechanism (PIS‐Unet), and observe its effectiveness in pancreatic tumor images segmentation, thereby providing supportive recommendations for clinical practitioners.

**Materials and methods:**

A total of 303 patients with histologically confirmed pancreatic cystic neoplasm (PCN), including serous cystic neoplasm (SCN) and mucinous cystic neoplasm (MCN), from Shanghai Changhai Hospital between March 2011 and November 2021 were included. A total of 1792 T2‐weighted imaging (T2WI) slices were used to build a CNN model. The model employed a pyramid pooling Inception module with a fused attention mechanism. The attention mechanism enhanced the network's focus on local features, while the Inception module and pyramid pooling allowed the network to extract features at different scales and improve the utilization efficiency of global information, thereby effectively enhancing network performance.

**Results:**

Using three‐fold cross‐validation, the model constructed by us achieved a dice score of 85.49 ± 2.02 for SCN images segmentation, and a dice score of 87.90 ± 4.19 for MCN images segmentation.

**Conclusion:**

This study demonstrates that using deep learning networks for the segmentation of PCNs yields favorable results. Applying this network as an aid to physicians in PCN diagnosis shows potential for clinical applications.

## 1 INTRODUCTION

Pancreatic cancer is a highly lethal disease. According to GLOBOCAN estimates, pancreatic cancer was the seventh leading cause of cancer‐related deaths worldwide in 2018, with approximately 432 000 deaths out of 459 000 cases.^1^ Due to the lack of symptoms in the early stages, abdominal pain, nausea, and other general symptoms are often attributed to other diseases, leading to delayed diagnosis of pancreatic cancer.^2^ Related studies have shown that the median age of pancreatic cancer diagnosis in the United States is 71 years, with only about 20% of cases detected before the age of 60.^3^, ^4^ Furthermore, the difficulty in detecting small lesions in the pancreas, the challenging access to the organ, its small size, and variable shapes, as well as the limitations of conventional imaging methods, contribute to over half of pancreatic cancers being diagnosed at an advanced stage, with a low survival rate of only 7%.^5^, ^6^, ^7^ Pancreatic cystic neoplasms (PCNs) are tumor‐like cystic lesions in the pancreas, including serous cystic neoplasm (SCN) and mucinous cystic neoplasm (MCN), among others. SCN is a benign tumor, and its management primarily involves observation and follow‐up. MCN is considered a precancerous lesion of the pancreas, carrying a certain risk of malignancy, and generally requires surgical resection.^8^ The treatment strategies for different categories of PCNs are markedly different. Therefore, it is crucial, especially in the early stages of PCNs, to utilize imaging information to determine the tumor category, accurately distinguish between benign and malignant tumors, and promptly devise subsequent plans, which is of significant importance in improving patient survival rates

Due to the limited effectiveness and reliability of relying solely on patients' clinical presentations for PCN detection and diagnosis, the acquisition of pancreatic imaging information through medical imaging techniques plays a crucial role in the auxiliary diagnosis of pancreatic tumors.^9^ Currently, CT, MRI, and EUS are commonly used imaging techniques for the detection of PCNs in clinical practice. Among them, CT is the most frequently utilized diagnostic modality due to its low cost. MRI, on the other hand, provides excellent soft tissue contrast and offers superior imaging of soft tissues. Both CT and MRI exhibit high detection rates for pancreatic cancer, with CT demonstrating slightly higher sensitivity compared to MRI, respectively reaching up to 96% and 93.5%.^10^ However, relevant studies have indicated that MRI has a higher detection rate for small masses and lesions with similar densities as shown on CT scans.^11^ Although conventional imaging methods can provide valuable pancreatic tumor imaging information, the accuracy of diagnosis heavily relies on the experience of radiologists who perform manual image interpretation. As a result of subjective factors associated with physicians, misdiagnosis and missed diagnosis are not uncommon occurrences.

In recent years, with the advancement of artificial intelligence, deep learning techniques have been widely applied in the processing of medical images. The introduction of CNNs has greatly facilitated tasks such as image segmentation and classification in medical imaging. Image segmentation is a common task in medical imaging, and accurate segmentation of PCN, combined with clinical information, can provide significant assistance in subsequent tumor characterization, treatment planning, and evaluation of treatment outcomes, thereby possessing important clinical value.

Currently, many researchers have focused on using deep learning to assist in the segmentation of pancreatic tumors, and the application of CNN‐based pancreatic tumor image segmentation has provided strong evidence for the detection and diagnosis of clinical pancreatic tumors. Li et al.^12^ proposed a segmentation model that integrates multiple modalities and scales by adding new modules, providing new ideas for the construction of subsequent multimodal models. The U‐Net^13^ architecture, consisting of encoders and decoders, has been widely used for image segmentation tasks. Its lightweight and simple structure make it highly adaptable, and numerous researchers have proposed improvements based on the U‐Net for segmenting pancreatic tumors, achieving improved segmentation performance. Ren et al.^14^ incorporated attention modules into the U‐Net structure, allowing the network to focus more on local regions. Jiang et al.^7^ used a densely connected convolutional structure to build a U‐Net model and incorporated deformable convolutional modules and long‐short‐term memory structures to extract features at different scales, thereby improving the segmentation accuracy of pancreatic tumors. The improved U‐Net models have also been widely applied to the processing of other medical images. Latif et al.^15^ incorporated Inception modules at each level of the U‐Net network, capturing features at multiple scales and achieving good segmentation results on brain tumor MRI images. Han et al.^16^ introduced residual modules into the U‐Net and replaced convolutions with competitive multiscale convolutions, resulting in excellent performance in the segmentation of liver and liver tumors. These models have been applied to different types of medical images and segmentation tasks, demonstrating the ability of CNNs to efficiently extract features from medical images and gradually becoming important tools in the field of medical image segmentation, providing doctors with more accurate and efficient means of image analysis and diagnosis.

In this study, our goal is to improve the U‐Net network and develop a model for the segmentation of MRI images for two types of PCNs: SCN and MCN. We aim to enhance segmentation accuracy and comprehensively evaluate the segmentation performance. The main contributions of this work are summarized as follows:

1. We propose a novel U‐Net network structure that integrates multiple modules for the segmentation of two types of PCNs: SCN and MCN.
2. We introduce a pyramid pooling module (PPM) between the encoder and decoder structures, concatenating feature maps of different sizes to include more contextual information in the output feature map.
3. By incorporating channel attention mechanisms and Inception modules, the network was enabled to extract features at different scales while significantly reducing the number of parameters during training, thereby reducing training time. The addition of attention enhances the network's focus on channel relationships and effectively improves its performance.

The rest of this paper is organized as follows: Section 2 provides a detailed description of the proposed method. Section 3 describes the dataset preprocessing, parameter settings, evaluation metrics, and experimental results. Finally, Section 4 presents a summary and discussion of the research content.

## 2 MATERIALS AND METHODS

According to the research design, this study proposes a network model for pancreatic tumor segmentation. The data processing workflow can be divided into three main steps. Firstly, the identification of Regions of Interest (ROI) is performed on the original images in the dataset, and preprocessing is applied to both the images and labels. Then, the ROIs are cropped to extract images containing only the tumor regions. Finally, the images are divided into training and testing sets, which are inputted into the network for training to obtain a segmentation model, and the testing set images are segmented.

### 2.1 Patient population

The data for this study was obtained from patients with PCN admitted to Shanghai Changhai Hospital between March 2011 and November 2021. Scans were performed using GE Signa HDxt MR750. The patients were scanned in a supine position with phased‐array receiver coils covering the upper abdomen, from the diaphragm to the lower margin of the iliacus muscle. The imaging protocol included breath‐hold single‐shot fast spin‐echo T2WI with the following parameters: TR (repetition time) of 6,316 ms, TE (echo time) of 87 ms, matrix size of 224×270, FOV (field of view) of 360 mm×420 mm, slice thickness of 5 mm, and interslice gap of 1 mm. The inclusion and exclusion criteria for the study were as follows: 1) T2WI images with clear visualization of the tumor region and without artifacts, 2) complete diagnostic information and confirmation of either SCN or MCN by pathological examination, 3) patients combined with other types of pancreatic tumors are not included. Ultimately, a total of 303 patients were included in the study, including 206 with SCN and 97 with MCN, resulting in a collection of 1792 T2WI images (1085 for SCN and 707 for MCN).

Prior to inputting the images into the model for segmentation, several preprocessing steps were performed. First, 2D slice images were extracted from the original data of the 303 PCN patients and divided into SCN and MCN classes for segmentation experiments. To reduce interference from surrounding tissues in the images, the slice images were localized and cropped using annotated tumor label images, and then resized to a size of 512×512. Subsequently, the images were normalized and input into the network model for training, two types of image classes were trained separately on two models, and tests were conducted on the corresponding images. To validate the stability of the model, a three‐fold cross‐validation strategy was adopted, where the preprocessed image data was divided into training and test sets, with 20% of the training set used as a validation set. Finally, the model performance was evaluated based on the average metrics from the three‐fold cross‐validation.

### 2.2 PIS‐Unet network construction

In this study, the Unet architecture was selected as the backbone network for the segmentation of PCNs. The network consists of an encoder and a decoder. The encoder is responsible for extracting image features and includes four downsampling modules, each composed of two 3 × 3 convolutional layers and a 2 × 2 max‐pooling layer. The decoder upsamples the features extracted by the encoder and incorporates skip connections between layers of the same spatial size using concatenation. Convolutional layers are primarily used for feature extraction in this structure, while pooling layers are employed to reduce the size of parameter matrices and achieve downsampling.

**FIGURE 1:**
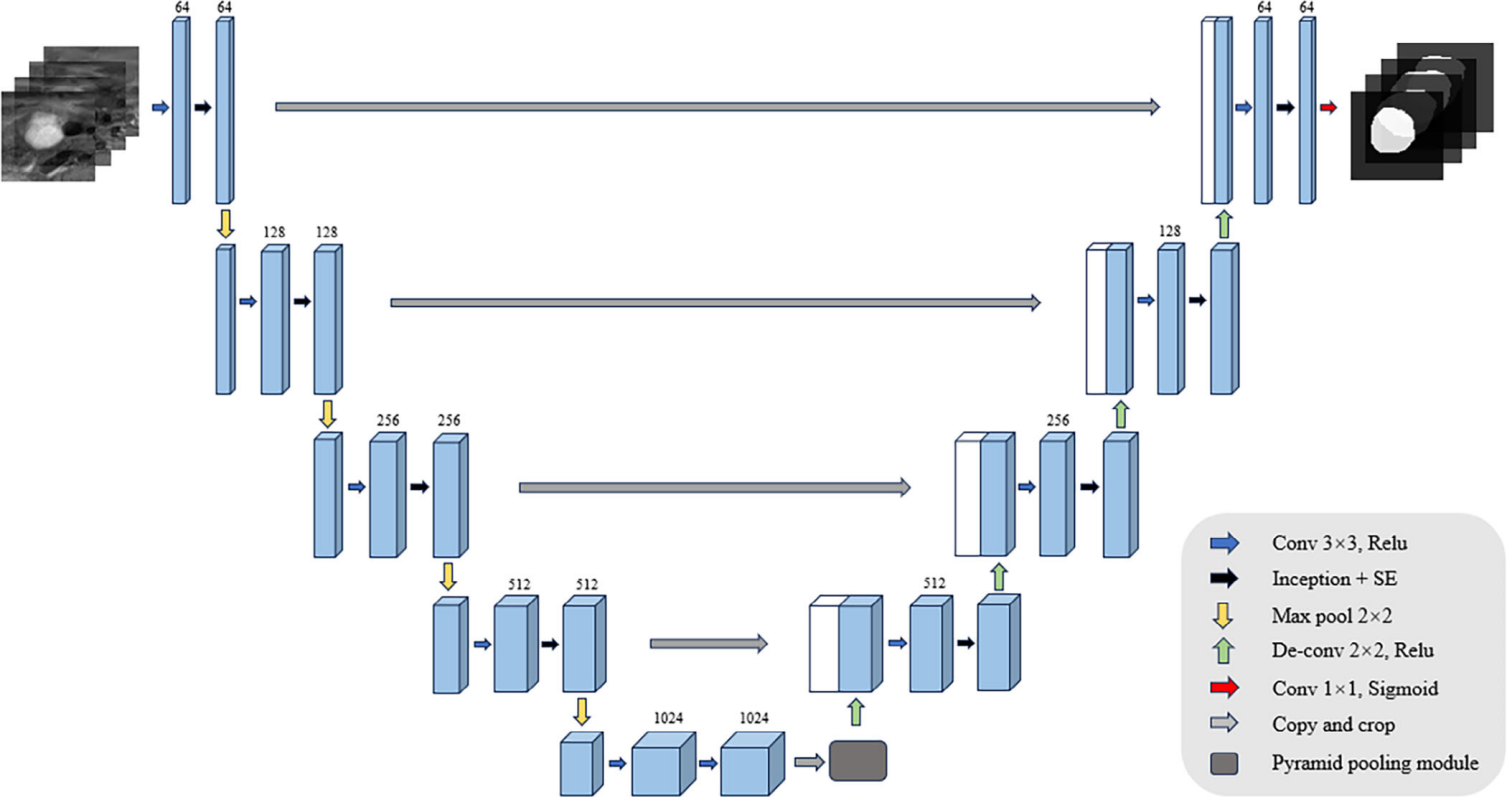
Overall architecture of pyramid pooling inception Unet with SE block.

To further emphasize the network's attention to local features, this study introduces several extensions to the original Unet architecture. First, an attention mechanism module is incorporated. Second, an Inception module is added before the attention mechanism module. Finally, a PPM is inserted between the encoder and decoder to further enhance the network's performance. By integrating the PPM, Inception module and SE attention mechanism, a pyramid pooling model with Inception architecture and SE attention mechanism (PIS‐Unet) is constructed, as shown in Figure 1. In this structure, the attention mechanism module enhances the network's focus on important features, the Inception module extracts multi‐scale features, and the PPM captures contextual information at different scales. The fusion of these components equips the PIS‐Unet model with stronger performance for pancreatic tumor segmentation tasks.

### 2.3 Fusion of I nception and SE modules

To improve the performance of the CNN model, this study adopts a strategy that combines the Inception and Squeeze‐and‐Excitation (SE) modules. Typically, increasing the depth and width of a network directly enhances its performance. However, this also leads to a sharp increase in the number of parameters. To address this issue, the Inception module is introduced. This module combines convolutional kernels of different sizes (1 × 1, 3 × 3, 5 × 5) and max‐pooling kernels (3 × 3). The 1 × 1 convolutional kernels are used for dimension reduction and parameter reduction, while the multi‐scale convolutional kernels effectively fuse features extracted at different scales.^17^ By parallelizing convolutional and pooling kernels of different sizes, extracting features from each size, and concatenating the outputs, a deeper feature matrix is formed.

The attention mechanism enables the network to focus on local features. Currently, there are attention mechanism modules that combine spatial and channel relationships, such as CBAM,^18^ as well as SE^19^ modules that focus on channel relationships. Since Unet incorporates skip connections between the encoder and decoder, it is necessary to enhance the network's attention to the relationships between image feature channels and adapt to the scarcity of medical image samples. Therefore, in this study, a lightweight SE module is selected and added. The principle of the SE module is as follows:

First, the global average pooling is applied to compress the feature map *A* of size HxW. The calculation formula is as follows:

(1) $$z_{k}=\;F\;\left(A_{k}\right)=\frac{1}{H\;x\;W}\;\sum_{i\;=\;1}^{H}\sum_{j\;=\;1}^{W}A_{k}\left(i,j\right)\;$$

In this formula, $A_{k}\left(i,j\right)\;$represents the value at the spatial position (i,j) of the feature mapA for the k‐th statistic, *H* and *W* represent the height and width of the feature map. Subsequently, the activation section, consisting of fully connected layers and activation functions, is applied to achieve the activation of the feature map. The calculation formula for the output feature vector *s* is as follows:

(2) $$s\;=\;Sigmoid\left(W_{2}\;RELU\left(W_{1}\;z\right)\right)$$

where $W_{1}\;$and *W*_2_ are weight matrices, Sigmoid is the activation function, RELU is the element‐wise activation function, and *z* represents the input feature vector. The first fully connected layer computes the weighted sum of the input feature vector *z* followed by an element‐wise activation function RELU. The second fully connected layer applies another weighted sum and activation function Sigmoid to obtain the output feature vector *s*. Finally, the scale module is applied to obtain the vector product $\tilde{\;x}$ of feature vector *s* and feature map *A*. The calculation formula for the vector product ${\tilde{\;x}}_{k}$ is as follows, where ${\tilde{\;x}}_{k}$ represents the vector product of channel *k*.

(3) $${\tilde{\;x}}_{k}=F_{scale}\;\;\left(z_{c},s_{c}\right)=z_{c}\;\;s_{c}\;$$

**FIGURE 2:**
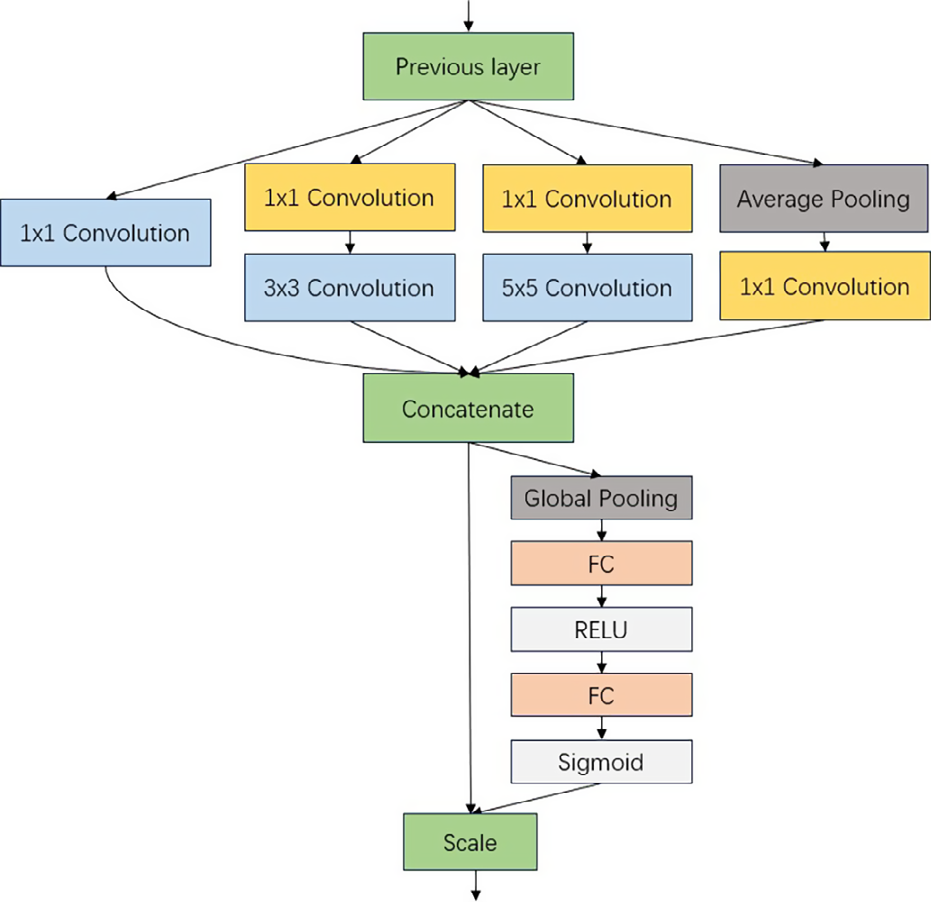
Overall structure of the Inception + SE module.

In this study, we replaced the latter 3 × 3 convolutional layer in each module of the basic Unet model with a composite module composed of a fusion of Inception and SE attention mechanisms. This concatenated structure not only reduces the number of parameters in the model but also allows the network to focus more on the channel features of the image and extract richer image features. In this structure, the Inception module is first used to perform multi‐scale convolution and pooling operations on the feature map to obtain features at multiple scales. This helps capture information from different receptive fields and enriches the representation of the features. Next, the SE module is applied to adaptively adjust the weights of each channel in the feature map. This attention mechanism allows the network to emphasize the channels that contribute more to the task of pancreatic tumor segmentation while suppressing less informative channels. By adjusting the channel weights, the network becomes more attentive to the relevant features. The adjusted feature map from the Inception + SE module is then passed to the convolutional layer of the next module for further feature extraction and processing. This integration of the Inception and SE modules enhances the capability of the network to capture and utilize multi‐scale and channel‐specific information, leading to improved performance in pancreatic tumor segmentation tasks. The complete structure of the Inception + SE module can be referred to in Figure 2, which illustrates the architecture and connections within the module.

### 2.4 Pyramid pooling module

**FIGURE 3:**
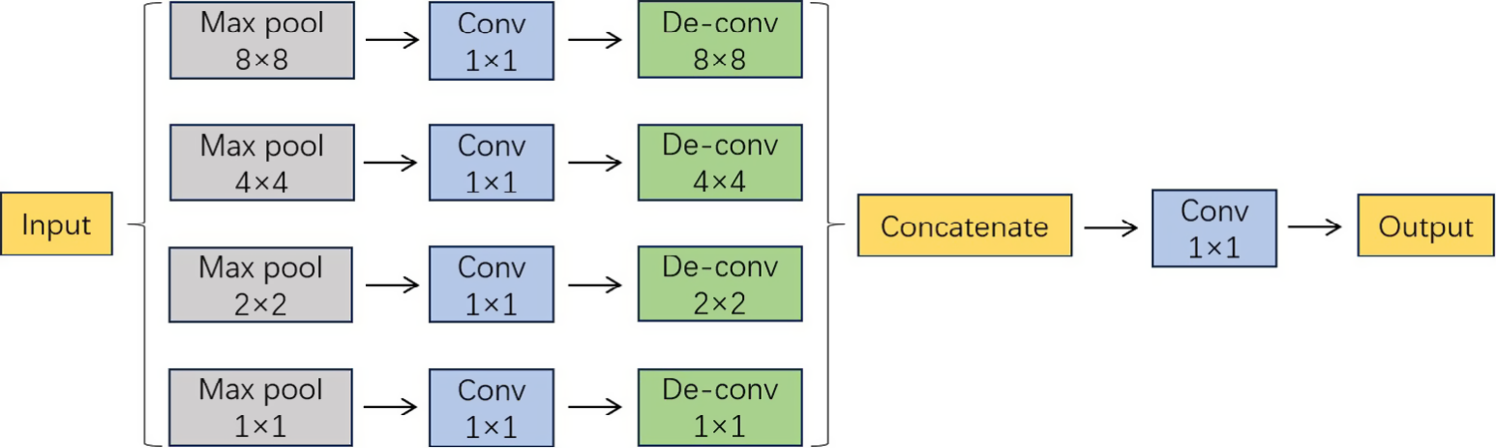
Overall structure of pyramid pooling module.

The PPM is introduced in this study between the encoder and decoder of the Unet model to further enhance the utilization efficiency of image information. Similar to the feature map concatenation principle in Unet, the PPM effectively extracts features at different scales and concatenates them to obtain more comprehensive contextual information, thereby improving segmentation accuracy. Specifically, the PPM performs pooling operations on the image using pooling kernels of different sizes, which results in feature maps of different scales. Each pooling kernel performs downsampling on the image to capture feature information at different scales. Then, a convolution operation is applied to each downsampled feature map to reduce its dimensionality and perform upsampling. Finally, all the feature maps at different scales are concatenated, and a 1 × 1 convolution is used to further reduce the dimensionality and obtain the final output feature map. The output feature map from the PPM has the same size as the input feature map but contains richer contextual information. This module enables the network to capture and utilize features at multiple scales, improving the overall understanding of the image and enhancing the segmentation performance. The complete structure of the PPM can be referred to in Figure 3.

### 2.5 Evaluation metrics

In order to assess the performance of the proposed PIS‐Unet model, five evaluation metrics were introduced: Dice Similarity Coefficients (DSC), Volumetric Overlap Error (VOE), Relative Volume Difference (RVD), Precision (PRE), and Recall. The calculation formulas for these metrics are as follows:

(4) $$DSC\;=\;2*\frac{U_{seg}\cap U_{gt}}{U_{seg}+U_{gt}}\;$$

(5) $$VOE\;=\;2*\frac{\left|U_{seg}-U_{gt}\right|}{U_{seg}+U_{gt}}\;$$

(6) $$RVD\;=\left|\frac{U_{seg}}{U_{gt}}-1\right|\;*100\%\;$$

(7) $$PRE\;=\frac{U_{seg}\cap U_{gt}}{U_{seg}}\;\;$$

(8) $$Recall\;=\frac{U_{seg}\cap U_{gt}}{U_{gt}}\;\;$$

where $U_{seg}$ represents the number of pixels in the model's segmentation result, and $U_{gt}\;$represents the number of pixels in the image label.

### 2.6 Implementation details

Datasets generation were conducted using PyCharm 2020.1.1 software and the Windows 10 operating system (64‐bit) on a NVIDIA RTX3060 GPU. Calculations and model training were performed on a system equipped with eight NVIDIA RTX4090 GPUs running Linux 7.9.2009 with CUDA version 11.3. The implementation was done using the Keras library in Python 3.8 environment, with Tensorflow as the underlying backend. The number of training epochs was set to 30. The optimizer used was Adam, with a learning rate of 0.0001 and a momentum of 0.9. The channel reduction ratio for the SE module was set to 16, which means that the number of channels was reduced to 1/16 of the original number in the first fully connected layer. The PPM incorporated four different pooling kernel sizes: 1×1, 2×2, 4×4, and 8×8. During the training process, the model with the minimum loss value was saved as the final model for generating segmentation results.

## 3 RESULTS

### 3.1 Patient characteristics

Among the 303 patients (19.14% male) enroll in this study, the mean age was 51.45 ± 13.53 years, the mean body mass index was 23.16 ± 3.24 kg/m^2^, the mean cystic neoplasm size was 4.03 ± 2.33 cm. About 4.62% patients combined with pancreatitis and 7.59% patients combined with diabetes mellitus.

### 3.2 Ablation experiments

To verify the improvement of network segmentation performance by different modules, this study conducted ablation experiments for validation. In the ablation experiments, Unet was used as the baseline model, and various modules were combined for validation. A three‐fold cross‐validation method was employed for evaluation, and the combination of modules for each model is presented in Table 1. In each experiment, the parameter settings were kept consistent, and the model's performance was evaluated using the average metrics of the three‐fold cross‐validation.

**TABLE 1:** Addition of modules in ablation experiments.

Networks	SE	Inception	PPM
Unet			
SE‐Unet	√		
PPM‐Unet			√
IS‐Unet	√	√	
PIS‐Unet	√	√	√

**FIGURE 4:**
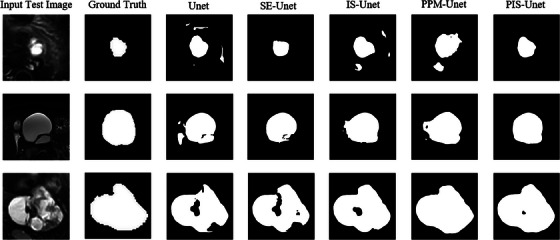
Segmentation results of 3 cases for MCN T2WI.

**FIGURE 5:**
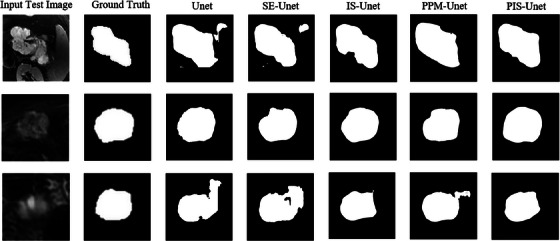
Segmentation results of three cases for SCN T2WI.

In the segmentation experiments on SCN and MCN images, the addition of different modules resulted in varying degrees of improvement in the network segmentation performance, with the PIS‐Unet model demonstrating the best performance. For SCN image segmentation, compared to the basic Unet network, the PIS‐Unet model increase 3.08% in DSC, a 3.91% increase in PRE, and a 1.01% increase in Recall. For MCN image segmentation, compared to the Unet model, the PIS‐Unet model increase 7.76% in DSC, a 3.42% increase in PRE, and a 8.07% increase in Recall. The comparative results of other relevant metrics can be found in Tables 2 and 3. The experimental results indicate that the attention mechanism, Inception module, and PPM play a certain role in improving network segmentation performance. Particularly, the PIS‐Unet model, which combines the attention mechanism, Inception module, and PPM in a pyramid pooling framework, exhibits the best segmentation results. The segmentation results of the models in the ablation experiments are shown in Figures 4 and 5.

**TABLE 2:** Comparison of segmentation performance of the ablation experiments for MCN.

Networks	DSC	VOE	RVD	PRE	Recall
Unet	80.14 ± 12.88	33.03 ± 24.43	30.60 ± 8.37	84.51 ± 2.10	82.26 ± 20.26
SE‐Unet	82.54 ± 13.08	28.57 ± 24.96	22.93 ± 13.32	87.87 ± 3.21	81.64 ± 18.40
PPM‐Unet	86.71 ± 4.54	21.66 ± 6.82	23.99 ± 2.77	85.14 ± 1.64	91.23 ± 9.86
IS‐Unet	85.05 ± 7.66	23.61 ± 12.18	24.51 ± 3.53	86.84 ± 1.78	87.28 ± 14.76
PIS‐Unet	87.90 ± 4.19	19.09 ± 5.47	21.06 ± 0.93	87.93 ± 2.93	90.33 ± 10.87

**TABLE 3:** Comparison of segmentation performance of the ablation experiments for SCN.

Networks	DSC	VOE	RVD	PRE	Recall
Unet	82.41 ± 2.75	27.73 ± 8.33	37.98 ± 16.45	80.13 ± 9.47	89.81 ± 6.42
SE‐Unet	83.73 ± 2.21	25.24 ± 4.32	29.13 ± 8.20	82.50 ± 6.79	89.10 ± 3.67
PPM‐Unet	84.61 ± 2.40	24.46 ± 6.31	30.17 ± 12.93	83.14 ± 9.16	89.94 ± 6.07
IS‐Unet	84.80 ± 2.63	23.79 ± 4.45	27.82 ± 8.74	84.85 ± 8.60	88.33 ± 4.49
PIS‐Unet	85.49 ± 2.02	23.77 ± 5.54	29.49 ± 10.48	84.04 ± 7.23	90.82 ± 4.85

## 4 DISCUSSION

Due to the complex morphology of the pancreas, pancreatic tumor segmentation poses challenges. Currently, a two‐stage segmentation approach from coarse to fine segmentation has been proven effective for pancreatic tumor segmentation.^20^ This approach involves annotating the images in advance, defining bounding boxes, and confining ROIs to obtain coarse segmentation results in the first stage. These results are then fed into a fine segmentation model to obtain the final segmentation of pancreatic tumors. Setting ROIs can increase the proportion of the tumor area in the training images. This reduces computational tasks unrelated to the target area, allowing the model to focus on extracting features from the ROIs. As a result, it can enhance the model's learning effectiveness, alleviates the computer's training burden, and ultimately significantly improves the accuracy of pancreatic tumors segmentation.

To demonstrate the advantages of the proposed model for pancreatic tumor segmentation, a comparison is made between the results obtained using the proposed method and those from other recent studies, as shown in Table 4. For example, Guo et al.^21^ combined Unet with LOGIS‐MOS to construct a 3D tumor segmentation model. This model achieved a DSC of 83.2 ± 7.8% and an RVD of 18.6 ± 17.4% for the segmentation of 51 cropped tumor region images from 15 subjects with the ground truth bounding box information available. Zhou et al.^22^ incorporated a deep supervision mechanism into a fully convolutional network (FCN) and included both a pancreas segmentation module and a tumor segmentation module based on the pancreas segmentation mask. This model achieved a DSC of 63.44 ± 27.71% for the segmentation of 131 contrast‐enhanced abdominal CT volumes, which increased to 77.92 ± 12.83% when a bounding box was included. Ren et al.^14^ introduced a bimodal structure into the attention‐based Unet and fused clinical features such as age and gender with EUS image features. It was trained on EUS images of 107 cases of pancreatic ductal adenocarcinoma (PDAC), pancreatic neuroendocrine neoplasms (pNEN), and solid pseudopapillary neoplasms (SPN). The overall DSC achieved was 75.52%. Li et al.^12^ combined inter‐modal adversarial learning and intra‐modal multi‐scale adversarial supervision to construct a multi‐modal and multi‐scale adversarial segmentation model (MMSA‐Net). It tested the model on 327 PDAC subjects with multiple types of images. For 67 cases with T1‐weighted image data, the segmentation achieved a DSC of 64.52 ± 19.53%, which increased to 73.47% when a bounding box was included, providing new insights into the fusion of multi‐modal data for the development of future multi‐modal network models.

**TABLE 4:** Comparison of existing computer‐aided pancreatic tumors segmentation methods.

Literature	Image	Subject	Method	DSC ± SD, %
Guo et al.^21^	CT	51 tumors	LOGIS‐MOS‐Unet	83.2 ± 7.8
Zhou et al.^22^	CT	131 tumors	FCN+deep supervision	77.92 ± 12.83
Ren et al.^14^	EUS	64 PDAC 28 pNEN 15 SPN	Attention‐Unet+clinical features	75.52
Li et al.^12^	MRI	67 PDAC	MMSA‐Net	64.52 ± 19.53
Ours	MRI	97 MCN 206 SCN	PIS‐Unet	85.49 ± 2.02 (SCN) 87.90 ± 4.19 (MCN)

The purpose of this study is to construct a network model that combines a pyramid pooling module with Inception architecture and SE attention mechanism for pancreatic tumor segmentation and accurately segment two kinds of PCNs. From the segmentation results shown in Figures 4 and 5, it can be observed that the basic Unet model struggles to accurately segment PCN, exhibiting severe over‐segmentation issues and producing rough segment boundaries for images with unclear borders. The addition of the SE module improves the issue of over‐segmentation to some extent but the segmentation performance remains suboptimal and can lead to under‐segmentation. Incorporating the Inception module on top of the SE‐Unet improves the handling of under‐segmentation and over‐segmentation issues, particularly for challenging samples with indistinct tumor borders, irregular shapes, and low contrast in the target region, the same issue also arises in the Unet network with only the PPM module added. Finally, adding the PPM module on top of the IS‐Unet further enhances the segmentation performance, achieving good segmentation results for most PCN images. Compared to other models, PIS‐Unet can segment tumor regions more comprehensively and produce smoother edge delineation, indicating its ability to extract features from PCN images more effectively. Overall, the model developed in this study exhibited a slightly better segmentation performance for MCN compared to SCN. This is primarily because deep learning focuses on image features. Due to differences in cyst fluid viscosity and the compactness of cystic components, MCN and SCN exhibit distinct variations in imaging features such as texture and grooves. SCN is commonly characterized by multi‐cystic lesions, making its image features more complex compared to MCN, hence resulting in a slightly lower segmentation outcome.

The pancreas is located deep within the abdomen, with significant variation in position and morphology. As a result, the morphology of acquired pancreatic tumor images tends to be irregular. Furthermore, some pancreatic tumors exhibit low contrast with surrounding healthy tissues in certain medical images. Challenges like the limited availability of public pancreatic tumor data further complicate segmentation tasks. To address these challenges, data augmentation can alleviate performance issues due to inadequate training data. Using multi‐modal image data can enhance tumor contrast with surrounding tissues, offering another viable approach to improve segmentation outcomes. In this study, we proposed a pyramid pooling Unet model that integrates attention mechanisms and Inception modules. The Unet serves as the base network, while the attention mechanism, Inception module, and PPMs are incorporated to improve the segmentation accuracy of various shapes of PCN images and provide assistance for subsequent diagnostic work. The SE attention mechanism enhances the network's focus on channel information, the Inception module captures features learned from convolutional kernels of different sizes, and the PPM enhances the utilization efficiency of global information. These modules work together synergistically to improve the performance of the segmentation model.

However, there are several limitations in our study. First, the dataset used only includes MRI images, while in clinical practice, CT and EUS are also commonly used for pancreatic tumor examination. Therefore, the generalization of the model needs further validation. Second, in the image preprocessing stage, manual annotation of tumor bounding boxes is required, and a fully automated segmentation process has not been achieved. Lastly, the validation of this study was conducted at a single medical center, and multi‐center data validation is crucial for the feasibility of future clinical applications.

## 5 CONCLUSION

The proposed pancreatic tumor segmentation model based on MRI images performs well on two types of PCN images, with a DSC of 87.90 ± 4.19 for MCN images and a DSC of 85.49 ± 2.02 for SCN images, and has great potential for specific clinical applications in pancreatic tumor segmentation. Future research can consider incorporating multi‐modal data to build a multi‐modal pancreatic tumor image segmentation model and validate it with data from multiple medical centers to improve the model's generalization. Additionally, further improvements can be made in tumor region detection algorithms to achieve fully automated segmentation of pancreatic tumors, ultimately providing stronger support and guidance for pancreatic tumor diagnosis and treatment research.

## AUTHOR CONTRIBUTIONS

Conceptualization and study design: Zhiwei Zhang. Data collection: Yun Bian, Hui Tian, and Zhenshun Xu. Statistical analysis and data interpretation: Jie Wu and Zhiwei Zhang. Manuscript preparation: Zhiwei Zhang. All authors have read and approved the final manuscript.

## CONFLICT OF INTEREST STATEMENT

The authors declare no conflicts of interest.

## Data Availability

Data available on request from the authors. The data that support the findings of this study are available from the corresponding author upon reasonable request.
